# Zwint-1 is required for spindle assembly checkpoint function and kinetochore-microtubule attachment during oocyte meiosis

**DOI:** 10.1038/srep15431

**Published:** 2015-10-21

**Authors:** Dong Woo Seo, Seung Yeop You, Woo-Jae Chung, Dong-Hyung Cho, Jae-Sung Kim, Jeong Su Oh

**Affiliations:** 1 Department of Genetic Engineering, College of Biotechnology and Bioengineering, Sungkyunkwan University, Suwon, Gyeonggi-do, 440-746, Korea; 2 Department of East-West Medical Science, Graduate School of East-West Medical Science, Kyung Hee University, Yongin, South Korea; 3 Division of Radiation Cancer Research, Korea Institute of Radiological and Medical Sciences, Seoul, Republic of Korea

## Abstract

The key step for faithful chromosome segregation during meiosis is kinetochore assembly. Defects in this process result in aneuploidy, leading to miscarriages, infertility and various birth defects. However, the roles of kinetochores in homologous chromosome segregation during meiosis are ill-defined. Here we found that Zwint-1 is required for homologous chromosome segregation during meiosis. Knockdown of Zwint-1 accelerated the first meiosis by abrogating the kinetochore recruitment of Mad2, leading to chromosome misalignment and a high incidence of aneuploidy. Although Zwint-1 knockdown did not affect Aurora C kinase activity, the meiotic defects following Zwint-1 knockdown were similar to those observed with ZM447439 treatment. Importantly, the chromosome misalignment following Aurora C kinase inhibition was not restored after removing the inhibitor in Zwint-1-knockdown oocytes, whereas the defect was rescued after the inhibitor washout in the control oocytes. These results suggest that Aurora C kinase-mediated correction of erroneous kinetochore-microtubule attachment is primarily regulated by Zwint-1. Our results provide the first evidence that Zwint-1 is required to correct erroneous kinetochore-microtubule attachment and regulate spindle checkpoint function during meiosis.

Reduction of chromosome number during meiosis is essential for producing haploid gametes from diploid parental cells. This reduction is achieved by two successive rounds of chromosome segregation, meiosis I (MI) and meiosis II (MII), after a single round of DNA replication. Although MII resembles mitosis in that sister chromatids separate and segregate to different daughter cells, the pattern of chromosome segregation during MI is unique. During MI, homologous chromosomes pair and then segregate from each other. Defects in this process result in aneuploidy, leading to miscarriages, infertility and genetic disorders such as Down’s syndrome^1^. Therefore, it is important to understand the molecular mechanisms that control chromosome segregation during meiosis.

The key step for faithful chromosome segregation is kinetochore assembly. Kinetochores are protein complexes that assemble on centromeric heterochromatin regions and mediate spindle attachment to chromosomes^2^. Kinetochores are composed of a number of conserved protein complexes, including the KMN network (Knl1, Mis12 and Ndc80) and the RZZ complex (Rod, Zw10 and Zwilch)^3^,^4^,^5^,^6^. The KMN network is primarily responsible for stable kinetochore-microtubule (kMT) attachment and recruitment of components of the spindle assembly checkpoint (SAC), a quality control mechanism that prevents anaphase onset until all chromosomes are stably attached to the spindle microtubules^3^,^6^. The functions of the KMN network are largely dependent on Aurora B kinase (in meiotic cells, Aurora C kinase functions in parallel with or substitutes for Aurora B kinase^7^,^8^,^9^,^10^), a component of the chromosome passenger complex (CPC)^11^. Aurora B kinase localizes to inner centromeres and breaks improper kMT attachments by phosphorylating multiple KMN components, which promotes correct kMT attachment at the kinetochores^3^,^12^. On the other hand, the RZZ complex has been shown to be essential for the recruitment of SAC proteins such as Mad1/2 and BubR1; thus, the RZZ complex is often classified as a SAC component^5^.

Recently, Zwint-1 was identified as an additional kinetochore-associated protein in a yeast two-hybrid screen for proteins that interact with Zw10^13^. Knockdown of Zwint-1 disrupts the localization of the RZZ complex to kinetochores^14^,^15^. More recently, Zwint-1 was shown to be phosphorylated by Aurora B kinase; this phosphorylation is required for the recruitment of the RZZ complex, which is in turn a prerequisite for the recruitment of SAC proteins^16^. Zwint-1 has also been found to interact with components of the KMN network^4^,^17^,^18^,^19^. Therefore, Zwint-1 appears to bridge the KMN network with the RZZ complex. This KMN-Zwint-1-RZZ connection enables signals to be properly transferred from Aurora B kinase, the main regulator of kinetochore assembly, to the SAC.

Although the functions of various kinetochore proteins in sister chromatid segregation during mitosis have been extensively studied, their roles in homologous chromosome segregation during oocyte meiosis are poorly understood. Here we investigated the function of the kinetochore protein Zwint-1 in oocyte meiotic maturation. We found that Zwint-1 is required for Aurora C kinase to correct erroneous kMT attachment and to activate the SAC at unattached kinetochores, thereby ensuring faithful segregation of homologous chromosomes during oocyte meiosis.

## Results

### Zwint-1 knockdown accelerates passage through meiosis I

**Figure 1:**
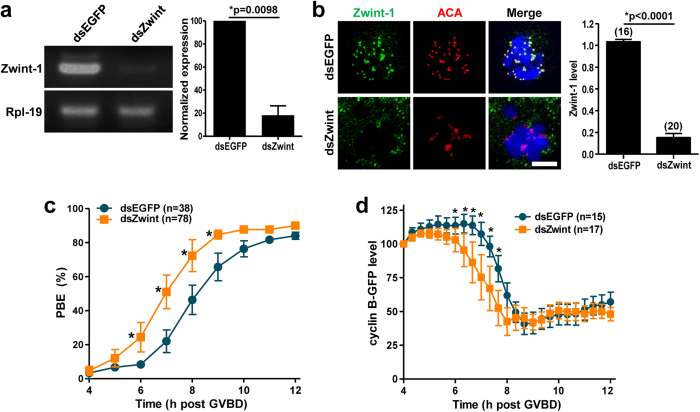
Zwint-1 knockdown accelerates polar body extrusion. GV oocytes injected with Zwint-1 dsRNA (dsZwint) were cultured for 13 h in the presence of IBMX, washed in IBMX-free medium, and allowed to progress to MetI. (**a**) RT-PCR of Zwint-1-depleted oocytes. Rpl-19 was used as a loading control. Normalized expression of Zwint-1 was quantified from two independent experiments and shown to the right panel (*p = 0.0098). (**b**) Immunostaining of Zwint-1-knockdown oocytes. Oocytes were fixed at 6–7 h after GVBD and immunostained with anti-Zwint-1 antibody. Kinetochores and DNA were stained with anti-centromere antibody (ACA) and DAPI, respectively. Shown are representative of three independent experiments with at least 30 oocytes. Scale bar, 10 μm. Quantification of Zwint-1 signals is shown in the right of images (*p < 0.0001). The number of oocytes analyzed is shown above the bars. (**c**) Timing of polar body extrusion (PBE) was determined in Zwint-1-knockdown oocytes. Data are mean ± SEM from three independent experiments with the indicated number of oocytes (*p < 0.05). The mean time at which half of oocytes completed PBE was 6 h 58 min for Zwint-1 knockdown and 8 h 5 min for control. (**d**) Oocytes depleted of Zwint-1 were injected with mRNA encoding cyclin B-GFP, and GFP levels were measured every 20 min up to 8 h. Quantification of cyclin B levels is shown. For each oocyte, the fluorescence intensity was normalized to the intensity recorded 4 h after GVBD. Data are mean ± SEM from one experiment with the indicated number of oocytes (*p < 0.05). Note that the cyclin B degradation was initiated around 7 h after GVBD for control and 6 h after GVBD for Zwint-1-knockdown oocytes.

To determine the function of Zwint-1 in meiosis, we microinjected long double-stranded RNA (dsRNA) targeting Zwint-1 into germinal vesicle (GV) stage oocytes and cultured them for 13 hours in the presence of 3-isobutyl-1-methylxanthine (IBMX) to block GV breakdown (GVBD). The level of Zwint-1 mRNA was significantly decreased by dsRNA injection (Fig. 1a). Knockdown of Zwint-1 on the protein level was also confirmed by immunostaining (Fig. 1b). This result also demonstrates that Zwint-1 is localized at the kinetochore during meiosis, consistent with the observations in somatic cells^13^,^15^,^18^,^19^. Zwint-1-knockdown oocytes underwent polar body extrusion (PBE), with no significant differences observed between control and knockdown oocytes (Fig. 1c). However, the timing of PBE was accelerated by 1 hour in Zwint-1-depleted oocytes compared with the control oocytes, suggesting that depletion of Zwint-1 results in precocious anaphase onset, followed by premature PBE. Given that anaphase onset and subsequent PBE are regulated by anaphase-promoting complex/cyclosome (APC/C)-mediated cyclin B degradation^20^,^21^, we monitored the cyclin B level after injection of mRNA encoding cyclin B-GFP. Consistent with the observed premature PBE, cyclin B levels also exhibited an early decrease in Zwint-1-knockdown oocytes (Fig. 1d), indicating accelerated passage through MI.

### Acceleration of meiosis I is due to a failure to recruit Mad2 at kinetochore in Zwint-1-knockdown oocytes

**Figure 2:**
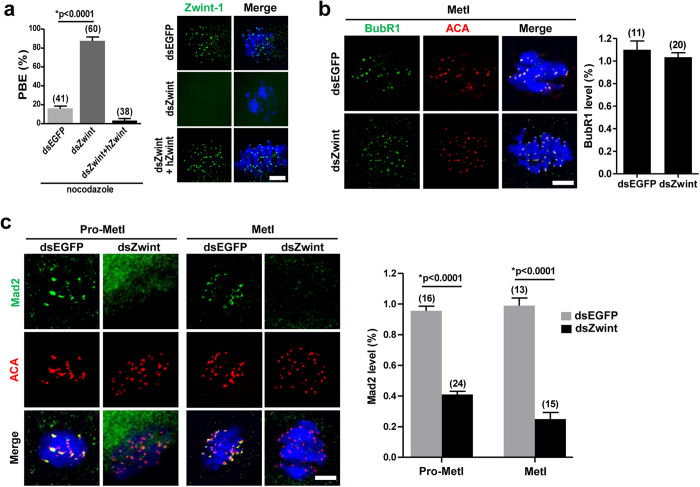
Mad2-mediated SAC inactivation in Zwint-1-knockdown oocytes. (**a**) Oocytes injected with the indicated dsRNA were cultured with 400 nM nocodazole from 4 h after GVBD and the PBE rate was scored at 13 h after GVBD. Data are mean ± SEM from three independent experiments (*p < 0.0001). The number of oocytes analyzed is shown above the bars. The exogenous expression of human Zwint-1 (hZwint) was shown by immunostaining in the right panel. Scale bar, 10 μm. Note that the PBE induced by Zwint-1 knockdown was rescued by overexpression of hZwint. (**b,c**) Zwint-1-knockdown oocytes were fixed at 2–3 h (Pro-MetI) or 6 h (MetI) after GVBD and immunostained with either anti-BubR1 (**b**) or anti-Mad2 (**c**) antibodies. Control oocytes injected with dsEGFP were fixed at 4 h or 8 h after GVBD corresponding to Pro-MetI or MetI, respectively. Kinetochores and DNA were stained with ACA and DAPI, respectively. Scale bar, 10 μm. Quantification of fluorescent intensity of BubR1 and Mad2 is shown in the right panel of images. Data are mean ± SEM of the indicated number of oocytes from two independent experiments (*p < 0.0001).

Since the SAC controls the timing of PBE by regulating APC/C activity^22^,^23^,^24^, we next asked whether the accelerated PBE in Zwint-1-depleted oocytes is a result of the failure to activate the SAC. To test this possibility, oocytes were cultured with nocodazole from 4 hours after GVBD to activate the SAC. While less than 20% of all oocytes exhibited PBE in the control group, about 85% of all Zwint-1-depleted oocytes showed PBE (Fig. 2a). This phenotype was specific to Zwint-1-knockdown, as it was rescued by overexpression of human Zwint-1 (Fig. 2a). To further confirm the inactivation of the SAC, the localizations of Mad2 and BubR1 were determined after Zwint-1 knockdown. Interestingly, BubR1 remained at kinetochores, while Mad2 no longer localized to kinetochores in Zwint-1-depleted oocytes (Fig. 2b,c). These results suggest that Zwint-1 selectively regulates the kinetochore recruitment of SAC proteins during meiosis, consistent with a previous report in somatic cells^19^. Therefore, our results demonstrate that Zwint-1 is required to localize Mad2 at kinetochores, but not BubR1.

### Abnormal chromosome alignment and high incidence of aneuploidy in Zwint-1-knockdown oocytes

**Figure 3:**
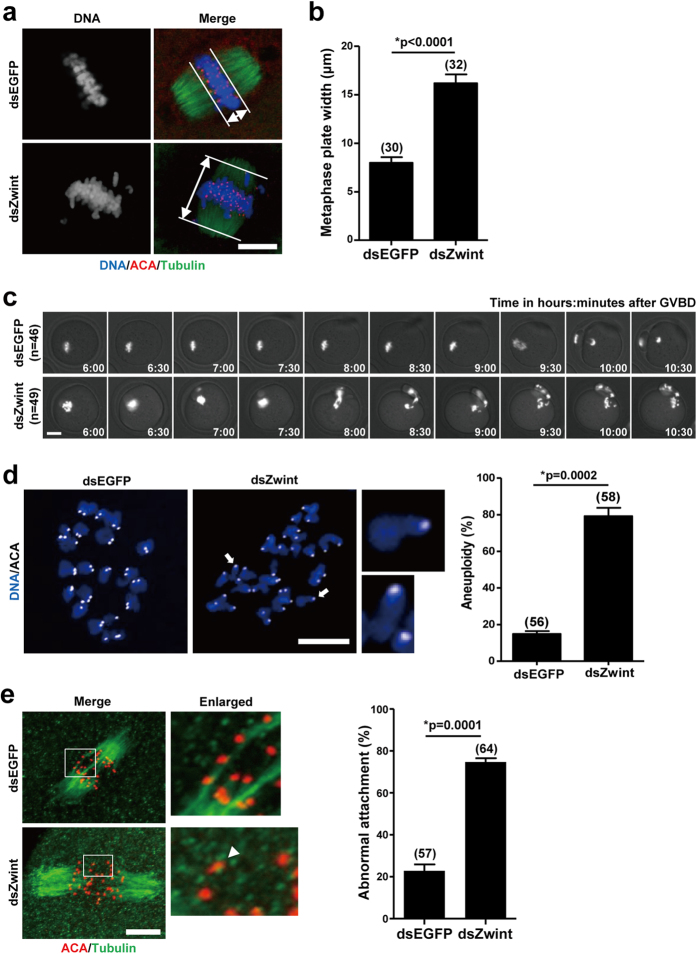
Chromosome misalignment and incorrect kMT attachment in Zwint-1-knockdown oocytes. (**a**) Oocytes in MetI (6 h after GVBD for Zwint-1-knockdown and 8 h after GVBD for control oocytes) were fixed and stained with anti-tubulin antibodies, ACA, and DAPI. Scale bar, 10 μm. (**b**) Metaphase plate width was determined by measuring the axis distance between the two lines at the edges of the DNA. Data are mean ± SEM from three independent experiments with the indicated number of oocytes shown above the bars (*p < 0.0001). (**c**) Control or Zwint-1-knockdown oocytes expressing histone H2B-mCherry were visualized by time-lapse microscopy. Snapshot images from a representative movie are shown. The indicated number of oocytes has been analyzed in three independent experiments. Scale bar, 20 μm. Note that the PBE was accelerated and chromosomes were misaligned in Zwint-1-knockdown oocytes. (**d**) Oocytes in MetII stage were cultured with 200 μM monastrol for 2 h and chromosome spreads were performed. Arrows indicate single chromatids enlarged. Scale bar, 10 μm. Quantification of aneuploidy is shown in the right panel. Data are mean ± SEM from three independent experiments (*p = 0.0002). The number of oocytes analyzed is shown above the bars. (**e**) Oocytes were cold-treated and stained with anti-tubulin antibodies and ACA at 8 h after GVBD for control and 6 h after GVBD for Zwint-1 knockdown. The area outlined in the white line is enlarged in the right panel. The arrow head indicates an unattached kinetochore. Scale bar, 10 μm. The unattached kinetochores were quantified in the right panel. Data are mean ± SEM from three independent experiments. The number of oocytes analyzed is shown above the bars (*p = 0.0001).

Given that defects in SAC activity increase the risk of aneuploidy^25^, we hypothesized that loss of Zwint-1 causes misaligned chromosomes during meiosis and, consequently, aneuploidy. To test this hypothesis, oocytes injected with dsRNA were cultured to the MetI stage and the positions of homologous chromosomes were determined. Whereas chromosomes were well-aligned at the metaphase plate in control oocytes, Zwint-1-knockdown oocytes exhibited an increased incidence of chromosome misalignment (Fig. 3a,b). To further confirm chromosomal defects in Zwint-1-knockdown oocytes, we imaged oocytes overexpressing histone H2B-mCherry (Fig. 3c and Supplementary Movie S1). In addition to premature PBE, homologous chromosomes were misaligned and separated improperly, showing lagging chromosomes. Moreover, sister chromatids remained misaligned during MetII stage following homologous chromosome segregation, suggesting the occurrence of aneuploidy in Zwint-1-knockdown oocytes. Indeed, we observed a high incidence of aneuploidy in Zwint-1-knockdown oocytes (Fig. 3d).

We next examined kMT attachment, since misaligned chromosomes and aneuploidy are often caused by inappropriate kMT attachment. Microtubules that are stably attached to kinetochores are cold-stable, whereas unattached microtubules are immediately disassembled after cold treatment^26^. Thus, we performed cold treatment with oocytes at the MetI stage and observed the resultant spindle morphology. Whereas distinct clustered kMTs were observed in control oocytes, unattached kinetochores and misaligned chromosomes were detected in Zwint-1-depleted oocytes (Fig. 3e). These results suggest that Zwint-1 is required for correct kMT attachment, thereby ensuring chromosome alignment on the metaphase plate during MI. Taken together, our data demonstrate that Zwint-1 is essential for accurate homologous chromosome segregation by regulating kMT attachment, since disruption of Zwint-1 function resulted in abnormal chromosome segregation and aneuploidy.

### Zwint-1 is required for chromosome alignment and kMT attachment independently of Mad2

**Figure 4:**
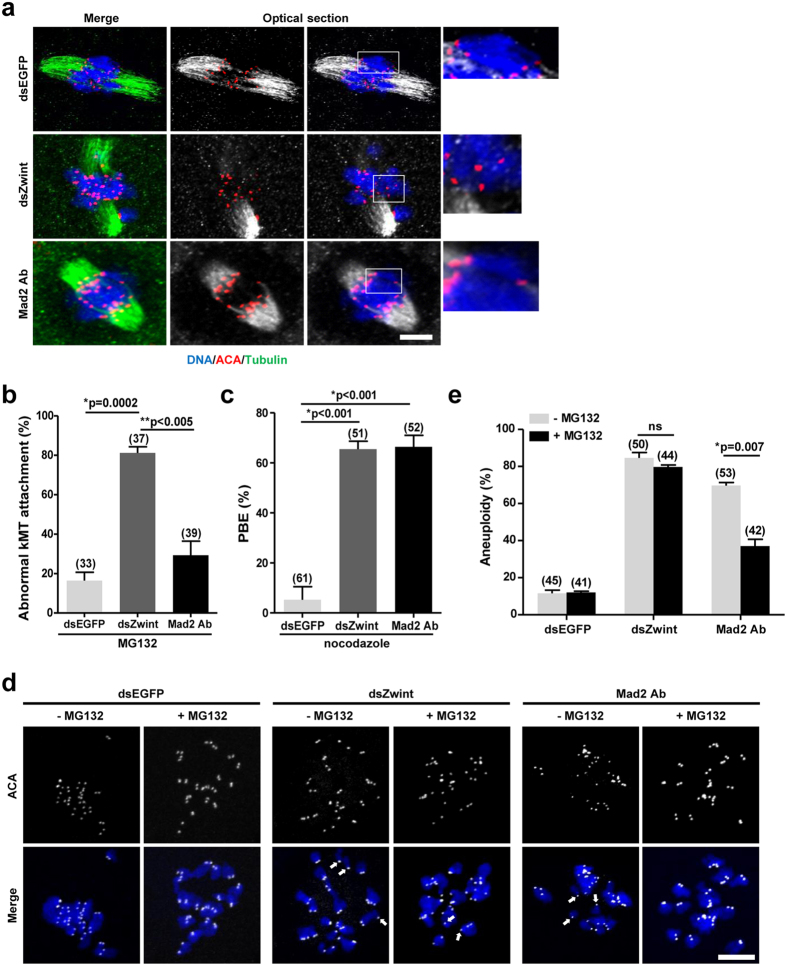
Chromosome misalignment and abnormal kMT attachment are not repaired in Zwint-1-knockdown oocytes. (**a**) Oocytes injected with either Zwint-1 dsRNA (dsZwint) or anti-Mad2 antibodies (Mad2 Abs) were treated with 25 μM MG132 at 4 h after GVBD. After 6 h culture, oocytes were cold-treated and immunostained with anti-tubulin antibodies, ACA and DAPI. The representative images from two independent experiments are shown. The area outlined in the white line is enlarged in the right panel. Scale bar, 10 μm. (**b**) Abnormal kMT attachments were scored. Data are mean ± SEM from two independent experiments (*p = 0.0002; **p < 0.005). The number of oocytes analyzed is shown above the bars. (**c**) Oocytes injected with either Zwint-1 dsRNA (dsZwint) or anti-Mad2 antibodies (Mad2 Abs) were cultured with 400 nM nocodazole from 4 h after GVBD and the PBE rate was determined at 13 h after GVBD. Data are mean ± SEM from three independent experiments (*p < 0.001). The number of oocytes analyzed is shown above the bars. (**d**) Oocytes were treated with (+) or without (−) MG132 at 4 h after GVBD and cultured for 6 h. Oocytes were matured to MetII stage by releasing from MG132-mediated inhibition and cultured with monastrol for 2 h prior to chromosome spreads. Arrows indicate the unpaired chromatids. Scale bar, 10 μm. (**e**) Quantification of aneuploidy. Data are obtained from three independent experiments with the indicated number of oocytes shown above the bars. (*p = 0.007; ns, not significant).

Mad2 delays the onset of anaphase, allowing time for error correction^27^,^28^. Since Zwint-1 knockdown accelerated the passage through meiosis I due to the failure of Mad2 recruitment at kinetochores, we speculated that the chromosome misalignment induced by Zwint-1 knockdown is a result of insufficient time to repair erroneous kMT attachment as a consequence of Mad2 deficiency. To test this hypothesis, oocytes were cultured with MG132, a proteasome inhibitor, to delay anaphase onset, thereby allowing more time for error correction. kMT attachment was then assessed after cold treatment. Importantly, lagging chromosomes were still observed in Zwint-1-knockdown oocytes, whereas chromosomes were well-aligned in oocytes injected with anti-Mad2 antibodies (Fig. 4a,b). Moreover, the spindles were clustered and persisted after cold treatment in oocytes injected with anti-Mad2 antibodies, whereas Zwint-1-knockdown oocytes exhibited faint arrays of microtubules and unattached kinetochores (Fig. 4a). This observation is not an off-target effect of antibody injection, because antibody-injected oocytes were able to overcome nocodazole-mediated MetI arrest (Fig. 4c). Similar results were obtained when either Zwint-1 or Mad2 was depleted using siRNA, further supporting that chromosomal defects in Zwint-1 knockdown oocytes are not simply due to Mad2 deficiency (Supplementary Fig. S1).

To assess the consequences of prolonged MI, oocytes were released from MG132-mediated inhibition and the rates of aneuploidy were determined. Consistent with the observations that prolonging the duration of MI rescued Mad2 inhibition-induced defects but did not restore Zwint-1-knockdown-mediated defects in chromosome alignment and kMT attachment, the aneuploidy rate was dramatically reduced in anti-Mad2 antibody-injected oocytes, whereas Zwint-1-knockdown oocytes still had a high incidence of aneuploidy (Fig. 4d,e). This result suggests that chromosome missegregation and aneuploidy induced by Mad2 inhibition are simply due to insufficient time for erroneous kMT attachment to be repaired. However, defects following Zwint-1 knockdown is not rescued by prolonging the duration of MI. Therefore, our data suggest that aneuploidy mediated by Zwint-1 knockdown is not a direct consequence of Mad2 deficiency, although the acceleration of MI after Zwint-1 knockdown is possibly dependent on the loss of SAC activity.

### Zwint-1 is required for Aurora C kinase to correct kMT attachment

**Figure 5:**
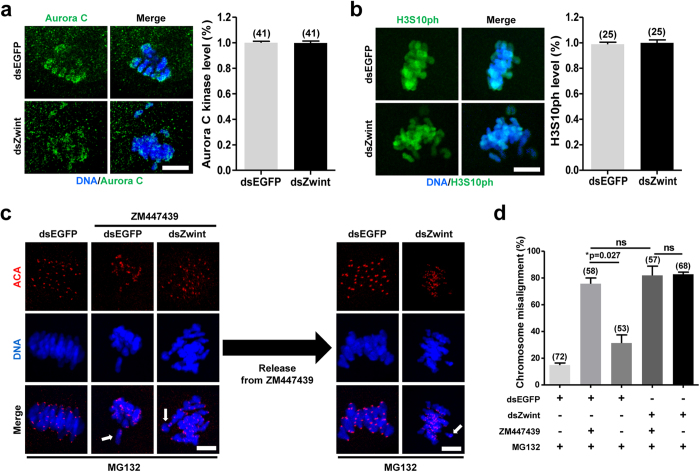
Zwint-1 is required for Aurora B/C kinase to correct chromosome misalignment during meiosis. Control or Zwint-1-knockdown oocytes were fixed at 8 h after GVBD or 6 h after GVBD, respectively, and immunostained with either anti-Aurora C kinase (**a**) or anti-H3S10 ph (**b**) antibodies. Scale bar, 10 μm. Quantification of fluorescent intensity is shown in the right panel of images. (**c**) At 4 h after GVBD, oocytes were cultured with 10 μM ZM447439 for 5 h. To prevent premature PBE, 25 μM MG132 was added in the culture media. Oocytes were then washed out from ZM447439 and cultured for 2 h in the presence of MG132. Oocytes were fixed and immunostained with ACA and DAPI. Scale bar, 10 μm. (**d**) Quantification of chromosome misalignment was shown. Data are obtained from three independent experiments with the indicated number of oocytes (*p = 0.027; ns, not significant).

Since Aurora B/C kinase activity is well-established to regulate kMT attachment and chromosome segregation^8^,^10^,^12^,^29^,^30^, we next asked whether inhibition of Zwint-1 affects Aurora B/C kinase activity, thereby producing defects in chromosome alignment and SAC activation. To this end, we examined the localization and activity of Aurora C kinase. Aurora C kinase activity was assessed by measuring the phosphorylation of histone H3 serine 10 (H3S10ph), one of the best-known substrates of Aurora B/C kinase. Notably, no significant differences were observed in either Aurora C localization or the level of phosphorylated H3S10 ph in Zwint-1-knockdown oocytes compared with control oocytes (Fig. 5a,b). This result suggests that impaired SAC activation and kMT attachment in Zwint-1-knockdown oocytes does not result from disrupted Aurora C kinase activity.

To investigate relationship between Zwint-1 and Aurora C kinase, oocytes were treated with Aurora B/C kinase inhibitor ZM447439 and chromosome alignment was examined. To prevent premature PBE caused by ZM447439 treatment, oocytes were cultured with MG132. Similar to Zwint-1 knockdown, the chromosome misalignment was observed in ZM447439 treated oocytes. Importantly, the defects were repaired when the oocytes were washed out from ZM447439 in oocytes injected with dsEGFP. However, the chromosome misalignment was not restored in Zwint-1-knockdown oocytes even after ZM447439 washout (Fig. 5c,d). These results imply that Zwint-1 is required for Aurora C kinase to correct chromosome misalignment during meiotic maturation.

## Discussion

Accurate chromosome segregation is essential for maintaining genomic integrity. One of the key steps in this process is kinetochore assembly. However, the functions of kinetochore proteins during oocyte meiosis have not been extensively studied. Here, we demonstrated that Zwint-1 plays a pivotal role in homologous chromosome segregation by regulating kMT attachment and the SAC during meiotic maturation.

We found that Zwint-1 knockdown accelerated anaphase I onset by abrogating the activation of SAC, leading to premature PBE. Interestingly, BubR1 was still localized to kinetochores in Zwint-1-knockdown oocytes, while Mad2 was not. Consistent with this observation, BubR1, but not Mad2, has been shown to remain at kinetochores in mitotic cells depleted of Zwint-1^19^. This result implies that the kinetochore localizations of BubR1 and Mad2 are differentially regulated during meiosis as in mitosis. This conclusion is supported by the finding that BubR1 localization does not depend on Ndc80, a component of the KMN network, whereas Mad2 is directly associated with Ndc80^31^,^32^. Therefore, it is likely that two independent pathways are present in mouse oocytes for recruiting SAC proteins to unattached kinetochores.

BubR1 was recently shown to undergo autophosphorylation in a process dependent on centromere-associated protein E (CENP-E). CENP-E is a kinesin-like microtubule motor protein that is essential for chromosome congression during mitosis^33^,^34^. Moreover, CENP-E-dependent BubR1 autophosphorylation at unattached kinetochores is required for accurate chromosome segregation and SAC activation^33^. In this regard, it is possible that Zwint-1 regulates CENP-E-dependent BubR1 phosphorylation instead of BubR1 localization. Thus, Zwint-1 knockdown disrupts BubR1 phosphorylation without affecting BubR1 localization, thereby inhibiting the faithful segregation of homologous chromosomes during meiosis. It will be interesting to determine the phosphorylation status of BubR1 in Zwint-1-knockdown oocytes in future studies. Given that chromosome segregation in female meiosis I is notoriously error-prone^22^,^23^,^27^, it is also possible that oocytes enter anaphase onset without full inactivation of the SAC. Indeed, oocytes with incorrect kMT attachment can exist simultaneously with an active APC and meiotic progression^35^,^36^,^37^,^38^.

Aurora B/C kinases play a pivotal role in the correction of erroneous kMT attachment and SAC activation^8^,^10^,^12^,^29^,^30^,^39^,^40^. Hence, Aurora B/C kinases have been considered to be at the top of the signaling cascade that ensures accurate chromosome segregation. Since the phenotype of Zwint-1 knockdown is similar to that of Aurora B/C inhibition, it is possible to hypothesize that Zwint-1 disruption affects the activity of Aurora C kinase, thereby impairing SAC activation and kMT attachment during meiosis. However, this is not the case, since neither the localization nor the activity of Aurora C kinase was affected by Zwint-1 knockdown. However, we do note that chromosomal defects following ZM447439 treatment were not restored after removing the inhibitor in Zwint-1-knockdown oocytes, whereas the defects was rescued after the inhibitor washout in the control oocytes. These data, along with the observation that Aurora C kinase activity remains intact in Zwint-1-knockdown oocytes, indicate that Zwint-1 is a key component for Aurora C kinase-mediated correction of erroneous kMT attachment during meiosis. In addition, the erroneous kMT attachment induced by Mad2-mediated SAC inactivation was rescued when the MI duration was prolonged. Therefore, it is likely that the SAC, at least Mad2 associated portion, is not directly involved in the error correction process. Instead, this SAC simply extends the duration of MI to allow sufficient time for enough error correction to take place before anaphase onset. However, it could not be generalized because several checkpoint proteins including BubR1 and Mps1 are required for kMT attachment^22^,^41^.

The incidence of aneuploidy has been shown to be dramatically increased in aged oocytes^42^. Although the exact molecular mechanisms underlying this phenomenon are not yet known, one compelling hypothesis is age-dependent decline in SAC activity. Consistent with this hypothesis, SAC deficiencies in oocytes from older women have been proposed to contribute to aneuploidy^43^. Given that chromosome misalignment induced by Mad2 deficiency is restored when MI duration is prolonged, extension of MI duration may reduce the aneuploidy rate in aged oocytes. However, if the level of Zwint-1 also decreases with oocyte aging, like other SAC proteins, Aurora C kinase-mediated error correction may also be impaired in aged oocytes. In this case, prolonged incubation of aged oocytes may not reduce the aneuploidy rate. Therefore, it will be interesting to examine the level of Zwint-1 in aged oocytes in future studies.

Collectively, our data suggest that Zwint-1 is a key component that delivers signals from Aurora C kinase, which is localized at the inner centromeres, to processes occurring at the outer kinetochores, such as the recruitment of SAC proteins and the correction of erroneous kMT attachment. We also showed that depletion of Zwint-1 impairs the primary function of Aurora C kinase, which is the repair of erroneous kMT attachment. Our results provide the first evidence that Zwint-1 is required for Aurora C kinase to regulate kMT attachment and SAC activation during oocyte meiosis.

## Methods and Materials

### Animals

All mice used in this study were 3- to 5-week-old CD-1 female mice (Koatech). Animals were maintained with food and water *ad libitum* under a 14-hour light/10-hour dark cycle. All procedures for mouse care and use were conducted in accordance with the guidelines and approved by the Institutional Animal Care and Use Committees of Sungkyunkwan University.

### Oocyte collection and culture

Ovaries were isolated from 4- to 6-week-old CD-1 female mice 46–48 hours after intraperitoneal injection of 10 IU of pregnant mare’s serum gonadotrophin (PMSG). Oocytes were collected from follicles and recovered in M2 medium supplemented with 200 μM 3-isobutyl-1-methylxanthine (IBMX) to prevent meiotic resumption. For *in vitro* maturation, oocytes were washed and cultured in IBMX-free M2 or M16 medium in a 5% CO_2_ atmosphere at 37 °C. All reagents and media were from Sigma, unless otherwise stated.

### RNA isolation and RT-PCR

Total RNA was extracted from oocytes using an RNeasy Plus Micro kit (Qiagen) followed by reverse transcription using a Sensiscript RT kit (Qiagen) according to the manufacturer’s instructions. PCR was performed using the following primers: Zwint-1, TGAAGGCCACATACATGG and GCCTCTTGACCTCTGCAG; Rpl-19, GCAAGCCTGTGACTGTCC and GGCGCAGGATCCTCATCC.

### Cloning and *in vitro* mRNA synthesis

The full-length cDNA sequence encoding human Zwint-1 was provided by the Korea Human Gene Bank, Medical Genomics Research Center, KRIBB, Korea. mRNA for microinjection was prepared *in vitro* using an mMessage mMachine kit (Ambion), polyadenylated, and then purified with a Nucleospin RNA clean-up kit (Macherey-Nagel). For double stranded RNA, PCR products were used as a template for *in vitro* transcription using a MEGAscript Kit (Ambion). The primers used were: EGFP, ATTAATACGACTAACTATAGGGAGAATGGTGAGCAAGGGCGAG and ATTAATACGACTCACTATAGGGAGAGCTCGTCCATGCCGAGAG; Zwint-1, ATTAATACGACTCACTATAGGGAGATGAGCAGAGCCCTGATGC and ATTAATACGACTCACTATAGGGAGATTGGCTGTAAGGGCTCGC.

### Microinjection

Approximately 5–10 pl dsRNA, siRNA or mRNA was microinjected into the cytoplasm of oocytes using a FemtoJet microinjector (Eppendorf) with a Leica inverted microscope (DMIRB) equipped with a micromanipulator (Narishige). After injection, oocytes were cultured for 13 hours in M2 or M16 medium containing IMBX. The oocytes were then transferred to fresh medium and cultured under mineral oil at 37 °C in an atmosphere of 5% CO_2_ in air. The small interfering RNAs (siRNAs) were purchased from a local company (Bioneer). The mixture of two siRNAs targeting either Zwint-1 or Mad2 was used. The siRNA sequences were as following: Zwint-1, CUAAUUCUCCAAGCUGAUU and UCAUGCUAACUUUGACAGU; Mad2, CUAAGAUCCGACAUUGAUA and GUGUUCUCCGUUCGAUCUA.

### Time-lapse imaging

Oocytes injected with histone H2B-mCherry or cyclin B-GFP mRNA were placed in a heated chamber and fluorescence images were acquired using a Nikon Eclipse Ti inverted microscope equipped with a CCD cooled camera (DS-Qi1Mc, Nikon). For quantification of cyclin B level, GFP fluorescence intensity was quantified using ImageJ software (National Institutes of Health).

### Nocodazole and cold treatment of oocytes

For nocodazole treatment, 400 nM nocodazole (Sigma) dissolved in dimethyl sulfoxide (DMSO) was added to the culture medium at the indicated time point; control oocytes were treated with an equivalent volume of DMSO.

For analysis of cold-stable microtubules, oocytes were incubated in ice-cold M2 medium for 10 min, as described previously^44^.

### Immunofluorescence analysis

Oocytes at specific stages were fixed in 4% paraformaldehyde for 10 min and permeabilized in phosphate buffered saline (PBS) with 0.1% Triton X-100 for 15 min. Oocytes were then incubated with primary antibodies followed by Alexa Fluor-conjugated 488 and 594 secondary antibodies (Jackson ImmunoResearch). DAPI was used for DNA counterstaining. At least 20 oocytes were examined for each group, unless otherwise stated. The antibodies used in this study were: anti-Zwint-1 (Abcam; 1:50), anti-BubR1 (Abcam; 1:50), anti-Mad2 (Santa Cruz; 1:50), anti-Aurora C kinase (Origene; 1:50), anti-H3S10ph (Millipore; 1:50), anti-acetylated α-tubulin (Sigma; 1:500) and anti-centromere (Antibodies Incorporated; 1:50).

For aneuploidy analysis, oocytes that had been pretreated for 2 hours in 200 μM monastrol (Sigma) were fixed and stained with the indicated antibodies, as described previously^45^.

### Image analysis

Images were acquired using a confocal laser-scanning microscope (LSM 700; Zeiss) equipped with a C-Apochromat 63 × /1.2 water immersion objective. Optical sections were obtained at 1 μm intervals and converted into maximum intensity projections. Data analysis was performed using ZEN 2010 LSM software (Zeiss) and ImageJ software (National Institutes of Health).

### Statistical analysis

Statistical analysis was performed with GraphPad Prism (GraphPad Software). Data are representative of at least three independent experiments unless otherwise specified. The significance of differences between groups was analyzed by Student’s *t*-test; *p* values <0.05 were considered statistically significant.

## Additional Information

**How to cite this article**: Seo, D.W. *et al.* Zwint-1 is required for spindle assembly checkpoint function and kinetochore-microtubule attachment during oocyte meiosis. *Sci. Rep.* **5**, 15431; doi: 10.1038/srep15431 (2015).

## Supplementary Material

Supplementary Information

Supplementary Movie
