# Supplementary material for: Zwint-1 is required for spindle assembly checkpoint function and kinetochore-microtubule attachment during oocyte meiosis
Source: Sci Rep. 2015 Oct 21;5:15431. doi: 10.1038/srep15431 (PMC4614028; doi:10.1038/srep15431)

Zwint-1 is required for spindle assembly checkpoint function and kinetochore-microtubule attachment during oocyte meiosis

Dong Woo Seo[1], Seung Yeop You[1], Woo-Jae Chung[1], Dong-Hyung Cho[2], Jae-Sung Kim[3*] and Jeong Su Oh[1*]

[1]Department of Genetic Engineering, College of Biotechnology and Bioengineering, Sungkyunkwan University, Suwon, Gyeonggi-do, 440-746, Korea

[2]Department of East-West Medical Science, Graduate School of East-West Medical Science, Kyung Hee University, Yongin, South Korea

[3]Division of Radiation Cancer Research, Korea Institute of Radiological and Medical Sciences, Seoul, Republic of Korea

*To whom correspondence should be addressed: ohjs@skku.edu; jaesung@kirams.re.kr

Figure S1. siRNA-mediated knockdown of Zwint-1 or Mad2 in mouse oocytes. (a, b) Oocytes injected with either Zwint-1 or Mad2 siRNA were cultured for 13 h in the presence of IBMX, washed in IBMX-free medium, and allowed to progress to MetI. Oocytes were then fixed and immunostained with anti-Mad2 or anti-Zwint-1 antibodies. Kinetochores and DNA were stained with ACA and DAPI, respectively. The representative images from two independent experiments are shown. Scale bar, 10 μm. Quantification of fluorescence intensity is shown in the right of images (*p<0.0001). The number of oocytes analyzed is shown above the bars. (c) Oocytes injected with indicated siRNAs were treated with or without 25 μM MG132 at 4 h after GVBD. After 6 h culture, oocytes were cold-treated and immunostained with anti-tubulin antibodies, ACA and DAPI. The representative images from three independent experiments are shown. The area outlined in the white line is enlarged in the right panel. Scale bar, 10 μm. Abnormal kMT attachments were scored and shown in the right panel of images. Data are mean ± SEM from two independent experiments. The number of oocytes analyzed is shown above the bars.

Supplementary Movie 1

GV oocytes coinjected with histone H2B-mCherry cRNA and dsRNAs corresponding to EGFP or Zwint-1 were incubated for 13 h and then examined by time-lapse microscopy from 6 h after GVBD. Frames were taken every 15 min up to 10.5 h after GVBD. The video is shown at 4 frames/sec.

Supplementary Fig. S1

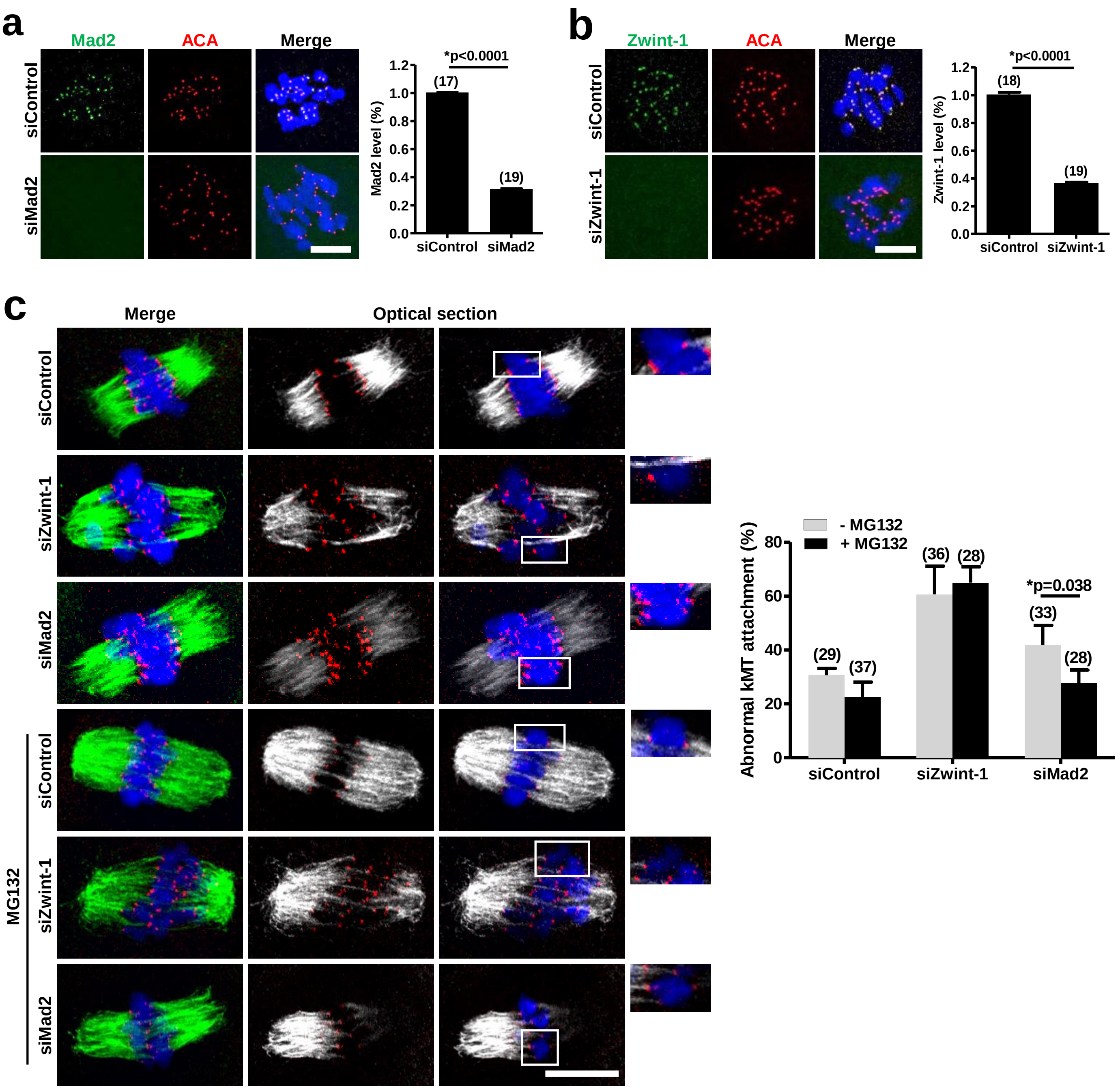

Supplement: Supplementary Information [file srep15431-s1.pdf]
